# Multiple Reprocessing of Conductive PLA 3D-Printing Filament: Rheology, Morphology, Thermal and Electrochemical Properties Assessment

**DOI:** 10.3390/ma16031307

**Published:** 2023-02-03

**Authors:** Mateusz Cieślik, Agata Rodak, Agnieszka Susik, Natalia Wójcik, Michał Szociński, Jacek Ryl, Krzysztof Formela

**Affiliations:** 1 Advanced Materials Center, Gdańsk University of Technology, Gabriela Narutowicza 11/12, 80-233 Gdańsk, Poland; 2 Institute of Nanotechnology and Materials Engineering, Faculty of Applied Physics and Mathematics, Gdańsk University of Technology, Gabriela Narutowicza 11/12, 80-233 Gdańsk, Poland; 3 Department of Polymer Technology, Faculty of Chemistry, Gdańsk University of Technology, Gabriela Narutowicza 11/12, 80-233 Gdańsk, Poland; 4 Department of Electrochemistry, Corrosion and Materials Engineering, Faculty of Chemistry, Gdańsk University of Technology, Gabriela Narutowicza 11/12, 80-233 Gdańsk, Poland

**Keywords:** multiple reprocessing, 3D-printing filament, PLA, conductive material, electrochemical properties, recycling

## Abstract

Additive manufacturing technologies are gaining more and more attention, resulting in the development or modification of 3D printing techniques and dedicated materials. On the other hand, economic and ecological aspects force the industry to develop material recycling strategies. In this work, the multiple reprocessing of a commercially available PLA conductive composite with carbon black filler, dedicated to 3D printing, was investigated. The effects of extrusion temperature (190 °C and 200 °C) and reprocessing steps (1–5 steps) on the rheology, morphology, thermal and electrochemical properties of the conductive PLA 3D-printing filament were evaluated. The results showed deterioration of the thermal stability and material strength, as well as the influence of reprocessing on the melting point, which increases after initial melting. The electronic conduction mechanism of the composite depends on the percolation paths and it is also affected by the multiple processing. The reversibility of the [Fe(CN)_6_]^3−/4−^ redox process diminishes with a higher degradation level of the conductive PLA. Importantly, the material fluidity was too high after the multiple reprocessing, which should be considered and suitably corrected during CB–PLA application as a 3D-printed electrode material.

## 1. Introduction

Fused deposition modeling (FDM^®^) or, more generally, material extrusion [1], is a 3D-printing technology that, due to the possibility of free structure design, is gaining popularity in many fields of industry such as the automotive [2], aviation [3] and the space industry [4]. The creation of new materials for 3D printing has also found application in the medical industry, where antibacterial filaments are created [5] or biocompatible filaments that can replace human bones [6], tissues or organs [7] are prepared. The filaments made of materials with high electrical conductivity has found particular interest in electronics. The commercially available conductive polylactic acid (PLA) filament is used to print electrical circuits and simple sensors such as temperature, stress and touch sensors [8]. In this field, the focus has been placed especially on the development of new conductive filaments that exhibit better mechanical properties while obtaining higher conductivity [9,10]. The conductivity of the conductive PLA-based filaments is due to carbon black (CB), which is a filler with a relatively low production cost that can be used in chemical analysis and electrochemical sensors. Moreover, CB can act as a UV absorber, donor/acceptor of free radicals, hydroperoxide decomposer and quencher of excited states [11]. Therefore, in specific conditions, CB fillers can prevent degradation of polymers, their blends or composites [12,13,14]. D’Urso et al. showed that a relatively small amount of CB stabilized the melt processing [15] of PLA, which can be beneficial during the preparation or recycling of PLA-based 3D-printing filaments.

Conductive carbon black can be easily combined with other materials such as metallic nanoparticles [16] or polymers [17]. Therefore, carbon black is employed to create the composite materials used for printing inks, as an active conductive component in polymers, pigments in paints or rubber reinforcements. In addition to the economic advantages of conductive carbon black related to its low cost, this material has fast charge-transfer kinetics and high analytical sensitivity. Thanks to this, the PLA matrix modified by carbon black or other carbonic fillers (e.g., carbon nanotubes, graphene, etc.) has found application in electrochemical measurements or sensors as filaments dedicated to 3D printing [18,19,20].

**Figure 1:**
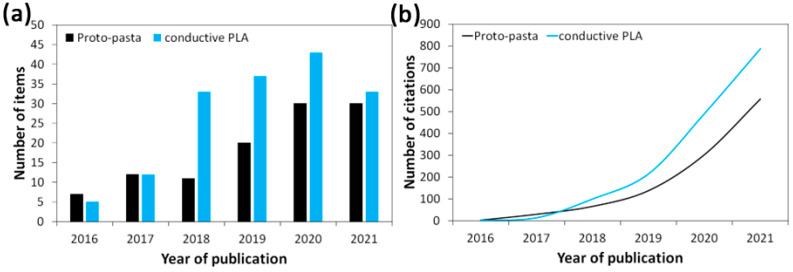
Trend of (**a**) number of scientific papers published; and (**b**) citation of works related to conductive PLA composites. Search was performed in the category “All Fields” in the period 2016–2021, considering the keywords “Proto-pasta” (the most popular conductive filament) and “conductive PLA”. Presented data were collected from the Scopus^®^ database on 5 October 2022.

Figure 1 summarizes the number of scientific papers published (Figure 1a) and citations (Figure 1b) related to conductive PLA composites. As can be observed, the interest in conductive PLA composites shows a dynamic growth, and current trends indicate further development of conductive filaments and their applications.

One of the advantages of additive technologies is the nearly maximum possible use of the raw material to create the object. Nevertheless, some material is wasted on supports, test prints, prototypes or unsuccessful prints that have no further use. The recycling of materials used in 3D printing is therefore a very important issue related to environmental protection. The close-looped recycling of waste polymers is a promising strategy for the sustainable development of 3D-printing technologies [21,22,23]. For this reason, more and more kits and solutions dedicated to the production and recycling of filaments—from home/laboratory conditions to the industrial scale—are appearing on the market.

At present, most studies related to the recycling of 3D-printing materials focus mainly on the aspect of the mechanical properties of these materials [24,25]. For example, Hong et al. [26] showed that the reprocessing of the PLA filament resulted in the deterioration of the material strength. During the tensile test, the sample was reprocessed three times, and a decrease in strength by 69% compared to the reference sample was observed. In the bending test, the trend of the results was similar: the strength of the sample reprocessed three times was lower by approx. 60% compared to the reference sample. One of the partial solutions proposed by Muñoz et al. [27] was to add from 20 to 40% of fresh PLA granules to the recycled material to obtain a good-quality filament.

Recently, the studies on the effect of thermal treatment and natural aging on the conductive PLA composites and 3D-printed sensors are gaining more and more attention [28,29,30]. This field of research allows a better understanding of the stability of 3D-printed materials as a function of their annealing conditions or environmental parameters. Moreover, this approach defines potential applications for prepared conductive PLA composites and shows the limitations of their performance properties, which can be considered as the routes for further development.

However, to the best of our knowledge, there is no information in the available literature about the impact of the multiple processing of commercial conductive filaments on their electrochemical characteristics. Therefore, the main aim of this work is to answer the question: How does the multiple reprocessing (from 1 to 5 times) of the commercially available conductive PLA 3D-printing filament affect not only the degradation of the polymer matrix, but also the electrochemical characteristics of the 3D-printed electrodes?

## 2. Materials and Methods

### 2.1. Materials

The material used for the research was the commercially available Proto-pasta conductive PLA filament with CB filler (CB–PLA). According to the specification provided by the producer, CB–PLA composition is the following: poly(lactic acid) >65%, carbon black <21.4%, and polymer (unknown composition—used probably as a plasticizer or an adhesive agent) <12.7%. The material was used as received. During storage, the filament and the samples were kept in sealed foil bags. Humidity of the materials was in the range of 0.09–0.19%, measured at 80 °C by the moisture analyzer MA 50./1.X2.IC.A (Radwag, Radom, Poland).

### 2.2. Sample Preparation

The material was remelted several times using the Mflow extrusion plastometer from ZwickRoell (Ulm, Germany). The filament was mechanically ground into pieces, 5 g of initial charge was measured, plasticized for 5 min, and passed through the plastometer from 1 to 5 times, at two temperatures: 190 °C and 200 °C. Used temperatures were selected based on general temperature range utilized during 3D printing of PLA. The load on the plastometer during the measurement was 10 kg.

After the third remelting, it was observed that the filament was not suitable for 3D printing. Therefore, for electrochemical measurement normalization, flat electrodes 40 mm × 10 mm and 2 mm thick were formed by pressure pressing at a temperature of 200 °C and a pressure of 10 MPa on the ZUP Nysa hydraulic press (Nysa, Poland). Pressing at elevated temperature lasted for 1 min. Then the mold was cooled for 5 min under a pressure of 10 MPa at ambient temperature.

### 2.3. Methodology

The melt mass-flow rate (MFR) and melt volume-flow rate (MVR) of the conductive PLA composites were measured as a function of reprocessing number, using the Mflow plastometer from Zwick/Roell (Ulm, Germany). The investigation was performed according to ISO 1133 (Procedures A and B) at 190 °C and 200 °C, with a load of 5 kg. Based on the MFR and MVR values, the density at 190 °C and 200 °C was also calculated using Equation (1):

(1) $$\rho_{m}=\frac{\mathrm{MFR}}{\mathrm{MVR}}$$

where *ρ_m_* is the melt density (g/cm^3^), MFR is the melt mass-flow rate (g/10 min) and MVR is the melt volume-flow rate (cm^3^/10 min).

Thermogravimetric analysis (TGA) was performed on the Netzsch TG 209 apparatus (Selb, Germany). The samples with approximate weight of 10 mg were placed in a corundum dish. The measurements were conducted in a nitrogen atmosphere for the temperature range 35–800 °C at a heating rate of 10 °C/min.

Differential scanning calorimetry (DSC) measurements were carried out under N2 with the Netzsch DSC 204 F1 Phoenix^®^ (Selb, Germany). The samples (ca. 6 mg) in the closed alumina crucibles were heated from 35 to 205 °C with a heating rate of 10 °C/min. From the DSC thermograms, the glass transition temperature (T_g_), melting temperature (T_m_), cold crystallization enthalpy (ΔH_cc_) and melting enthalpy (ΔH_m_) were evaluated using the Proteus 7.1.0 software. The presented values were determined from the first heating.

Scanning electron microscopy (SEM) studies were carried out using the FEI Quanta 250 FEG instrument, ThermoFisher Scientific, (Waltham, MA, USA) equipped with a Schottky field-emission gun, operating at an accelerating voltage of 30 kV. Before the examination, the samples were coated with nanometric layers of gold to increase their conductivity.

Raman spectroscopy measurements were performed under the ambient atmosphere with the LabRam Aramis spectrometer by Horiba Scientific Co., (Longjumeau, France) employing a laser of 532 nm wavelength. The spectral acquisition times were 5 scans accumulated with 10 sec/scan. The spectra were collected within the region from 200 to 3500 cm^−1^. Directly before the measurement, the filaments were broken in half to expose a fresh surface of the cross-section area for examination. For each filament, 5 spots distributed over the examined surface were tested to ensure the representativeness of the results.

The electrical properties of samples were studied using the broadband dielectric spectroscopy (BDS) technique. The measurements were conducted in the frequency range from 10 mHz to 1 MHz, with an AC voltage of 1 Vrms, using the Novocontrol Concept 40 broadband dielectric spectrometer Alpha-A (Montabaur, Germany), equipped with the ZG4 dielectric interface. The temperature range of tests was selected to coincide with the operation conditions of the materials and varied between 0 and 40 °C with a step of 5 °C for cooling and heating temperature. For the electrical measurements, gold electrodes were evaporated in vacuum at the plane parallel to the surfaces of the samples. The samples had a thickness close to 2 mm and a diameter close to 10 mm. The measurements were taken under a nitrogen atmosphere using the Quatro Cryosystem (Montabaur, Germany) temperature-controlling system.

Cyclic voltammetry (CV) studies were performed on the Gamry Reference 600+ potentiostat (Warminster, PA, USA). The electrochemical studies were carried out in a three-electrode setup, with CB–PLA as the working electrode, Ag|AgCl (3 M KCl) as the reference electrode and Pt mesh as the counter electrode, after initial conditioning for 10 min. The measurements were carried out in 1 mM [Fe(CN)_6_]^3−/4−^ in 0.1 M KCl solution. The CV scans were conducted at a 100 mV/s polarization scan rate. Before the electrochemical measurements, the electrode surface was electrochemically activated, using the procedure described in detail elsewhere [31].

## 3. Results and Discussion

### 3.1. Melting Rate of Reprocessed PLA-Based Filament

**Figure 2:**
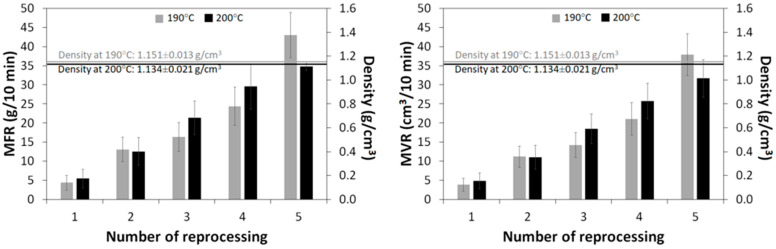
MFR, MVR and melt density of the conductive PLA 3D-printing filament as a function of reprocessing cycles.

MFR and MVR parameters are generally used for the primary determination of the processability and rheological behavior of molten thermoplastic polymers or polymer composites. The MFR and MVR values can also be correlated with the final properties of polymeric materials [32,33,34]. The MFR, MVR and melt density values of the conductive PLA 3D-printing filament as a function of the number of reprocessing cycles are presented in Figure 2.

As can be observed, the effect of consecutive reprocessing events on melt density was negligible. Moreover, as could be expected, the melt density of the PLA 3D-printing filament was slightly lower at a higher temperature. On the other hand, the MFR and MVR parameters increased significantly with the increasing number of reprocessing cycles. Surprisingly, the observed trend was higher for the MFR/MVR parameters determined at 190 °C, resulting in ~9.9 times growth after five reprocessing events, compared to ~6.5 times when reprocessed at 200 °C. This phenomenon can be explained by considering two factors affecting this measurement method. The first factor is related to the thermal degradation of the material during the measurement—in the studied case at 190 °C, the residence time of material inside the plastometer barrel was longer than at 200 °C. It was determined that, for the material extruded for the first time at 190 °C, the residence time in the plastometer was 11:39 min (5 min of preheating and 6:39 min of measurement), while for the material processed at 200 °C, the residence time in the plastometer was 9:21 min (5 min of preheating and 4:21 min of measurement). This could enhance the partial degradation of the conductive PLA 3D-printing filament.

The second factor is related to the presence of carbon black filler in the studied material, the distribution or orientation of which could change during each reprocessing cycle, thus affecting the melt flow rate. This will be discussed further when performing thermogravimetric analyses.

Wang et al. [35] investigated the effect of the melt flow rate of commercial PLA filaments on the fused deposition modeling of 3D-printed materials. The results showed that considering only the MFR parameter was insufficient for the proper assessment of the PLA-based filaments, because plasticizers and crystallinity degree also played an important role after the polymer melt deposition. Moreover, the authors recommended that the rheological properties estimated by MFR (or other suitable techniques) should be usually combined with detailed thermal and SEM analysis, which we present in the next sections of this paper.

### 3.2. Thermal Properties

**Figure 3:**
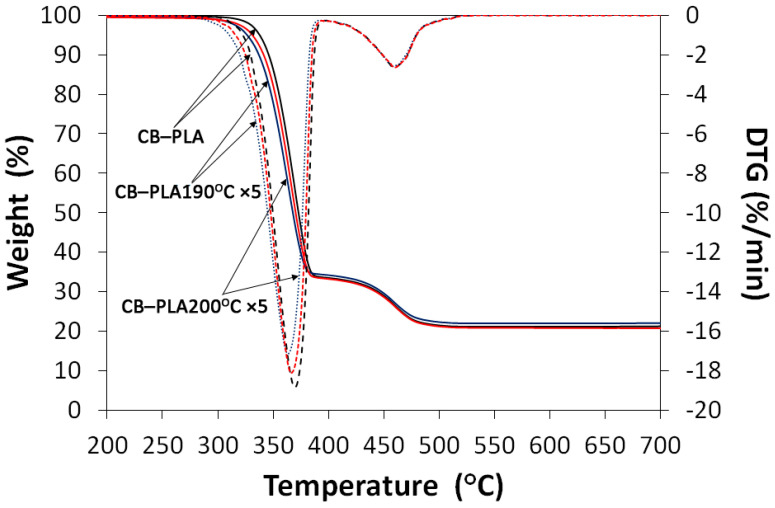
TGA and DTG curves for CB–PLA and CB–PLA ×5, reprocessed at 190 °C and 200 °C.

The thermal stability of the recycled conductive PLA 3D-printing filament was investigated by using the TGA and the derivative thermogravimetric (DTG) analyses. The obtained results are shown in Figure 3, while the decomposition temperatures as a function of weight losses (T_−2%_, T_−5%_, T_−10%_, and T_−50%_), maximum thermal degradation peaks (T_max1_, T_max2_) and char residue at 800 °C are summarized in Table 1.

For all materials, there are two thermal degradation steps. The first one with maximal degradation temperature T_max1_ ~ 365 °C is related to the decomposition of PLA [36], while the second one T_max2_ ~ 460 °C is probably from a polymeric additive used by the producer of the conductive PLA 3D filament. According to the technical specifications, the basic composition of the filament employed is the following: poly(lactic acid) >65%, carbon black <21.4%, and polymer (unknown composition—used probably as a plasticizer or an adhesive agent) <12.7%, which is in agreement with our results and the recently published papers [37,38].

Although the obtained results showed a similar trend for the studied materials, it should be noted that the reprocessing of the commercial conductive PLA 3D filament leads to the deterioration of its thermal stability. An increase in the number of reprocessing cycles led to a significant decrease in T_−2%_, T_−5%_, T_−10%_ and T_−50%_. The filament remelted at 200 °C showed better thermal stability than the materials processed at 190 °C, which is related to the residence time of the material in the barrel of the plastometer during reprocessing. For example, for the sample CB–PLA190 °C ×5, temperature T_−2%_ is 311.5 °C (15.7 °C lower than for the reference sample), while for CB–PLA200 °C ×5, T_−2%_ is 315.3 °C (11.6 °C lower than for the reference sample). A similar tendency was also observed for other degradation temperatures as a function of weight losses (T_−5%_, T_−10%_ and T_−50%_).

**Figure 4:**
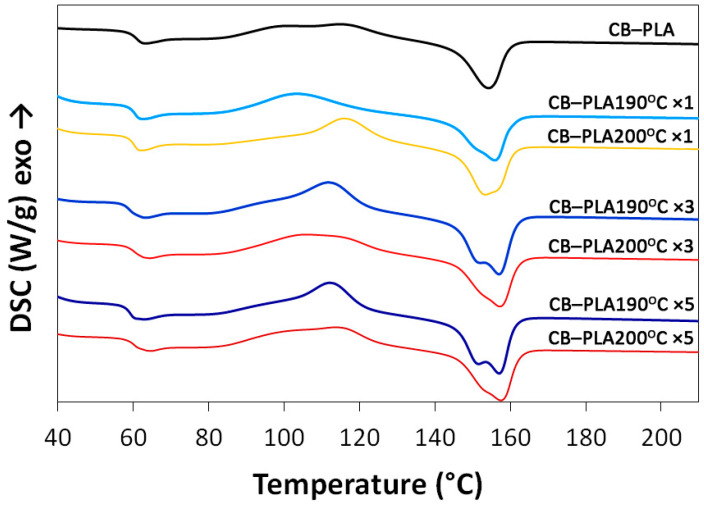
DSC measurements for studied materials.

Next, the thermal properties of the pure and reprocessed conductive PLA 3D filaments were analyzed using differential scanning calorimetry (DSC). The results were based on the heat released or absorbed when the material was heated. Figure 4 shows the DSC curves of the tested materials, while Table 2 summarizes thermal properties such as glass transition temperature (T_g_), melting temperature (T_m_), enthalpy of crystallization (ΔH_cc_), enthalpy of melting (ΔH_m_) and degree of crystallinity (χ_c_). It can be observed that T_g_ slightly decreased with the increasing number of reprocessing events for the samples processed at 190 °C, in contrast to the samples remelted at 200 °C. This could be due to the higher degradation of the polymer chains at the lower temperature, which is related to the longer residence time of the plastometer in the barrel. The degradation of polymer chains resulted in a facilitated movement of the PLA chains, which directly affected the glass transition temperature. It is noted that the number of melts also affects the formation of an exothermic cold-crystallization peak, which occurs at about 110 °C. This indicates the transformation of the amorphous phase contained in the material into a more ordered and crystalline structure. The crystallization enthalpy increases especially for the samples processed at a lower temperature, possibly due to the degradation of the material, which hinders the reorientation of the molecular chains. The melting point increases after the initial remelting and remains at a similar level after further processing. The heat of melting decreases after the first processing for both material types. The samples prepared at 190 °C show a higher increase in the enthalpy of melting during subsequent processing steps; in addition, an increase in the amount of remelting leads to the formation of two melting peaks in their case. This could be due to the degradation of the PLA chain or the polymer additive used by the producer, the composition of which is unknown.

For a better interpretation of the results obtained, Table 3 also presents the DSC results for conductive PLA-based materials described in the literature. As can be observed, for commercial CB–PLA (Proto-pasta conductive PLA), the glass transition temperature (T_g_) value is in the range 58.9–63.3 °C, average 61.1 ± 2.2 °C (this study: 58.7–60.2 °C). For comparison, PLA-based composites reinforced by CB (4–20 wt%) are characterized by T_g_ in the range 55–63.4 °C.

More visible differences can be observed for the melting temperature (T_m_) of conductive PLA-based materials described in the literature. For commercial CB–PLA (Proto-pasta conductive PLA), the T_m_ parameter is in the range 146.1–166.2 °C, average 149.1 ± 10.3 °C (this study: 153.4–157.9 °C). The melting temperature for PLA-based composites reinforced by CB (4–20 wt%) is in the range 166.7–178 °C.

PLA with CB filler (commercial product and lab-made material), regardless of the composition, showed T_m_ and T_g_ parameter values in the specific areas, which are affected by the characteristics of the components used (e.g., PLA molecular weight and structure, surface area and particles size of CB) and the melt-processing parameters, which result in the dispersion efficiency of the CB filler in the PLA matrix and also define matrix–filler interactions.

The differences in T_g_ and T_m_ parameters for commercial CB–PLA filaments described in the literature are related to the thermal treatment history of the studied materials. It should be mentioned that the thermal treatment of polymers, especially above their melting temperature, had a significant impact on the electrical conductivity of polymeric composites [39,40]. Therefore, this phenomenon should be considered while planning the experiments focused on lab-made conductive materials dedicated to 3D printing or sensing applications.

**Table 3:** DSC results for conductive PLA-based materials described in the literature.

Sample Composition	T_g_ (°C)	T_m_ (°C)	Heating Rate (°C/min)	Sample Weight (mg)	References
CB–PLA *	58.7–60.2	153.4–157.9	10	~6	This work
	58.9 ± 0.8	146.1 ± 0.5	3	~10	[41]
	63.3	152	-	-	[42]
	61.9	166.2	10	3	[43]
PLA + 5–15% CB	55–59	174–178	10	-	[44]
PLA + 4–20% CB	61.7–63.4	166.7–170.6	10	8–10	[45]

* Material manufactured by Proto-pasta—composition: PLA > 65%; CB > 21%; unknown polymer > 12.7%.

### 3.3. Surface Characterization

**Figure 5:**
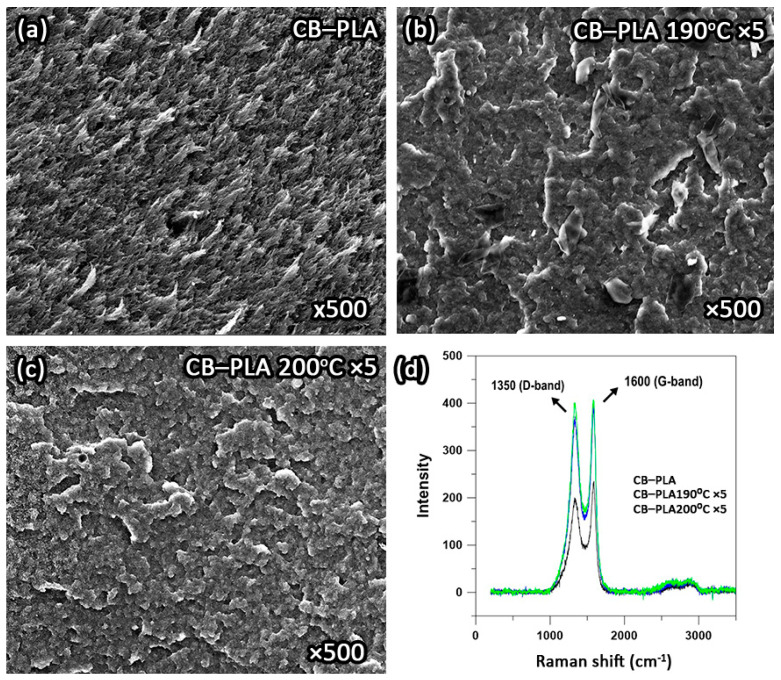
SEM micrographs of (**a**) reference sample, five-time reprocessed CB–PLA at (**b**) 190 °C, (**c**) 200 °C, and (**d**) Raman spectra of the pristine CB–PLA and after five reprocessing cycles.

The dispersion of fillers in polymer matrices has a significant effect on the morphology of composites. SEM was used to study the surface of conductive PLA composites. Figure 5a–c show the surface morphology of the reference sample and the five-time recycled CB–PLA at 190 °C and 200 °C. As can be seen from the attached SEM micrographs, the morphology of the CB–PLA and the samples remelted five times diverge. The surface of the untreated CB–PLA was significantly more fissured than that of the CB–PLA190 °C ×5 and CB–PLA200 °C ×5 samples. Large irregular depressions and protrusions indicate a higher agglomeration of the carbon black particles. Similar morphology was observed by Guo et al. [45], who investigated the effects of carbon black content on the conductivity and the mechanical as well as morphological properties of the CB–PLA composites. A higher content of carbon black (more than 12 wt.%) resulted in a worse dispersion in the PLA matrix.

The samples after reprocessing no longer show such an irregular structure. The surface is flatter, and no longer fissured. This is due to the fact that the samples processed at 190 °C and 200 °C degrade more after each reprocessing (because of the successive heating to high temperatures, annealing of the material and cooling), which affects the degradation of the polymer matrix chain. In turn, the degradation affects the rheological properties of the samples (as confirmed by the MFI measurements; see Section 3.1). A sample that has been degraded is characterized by a higher melt flow rate than a sample that has not been degraded. A more homogeneous surface of the samples after processing may also be due to a reduction in the number of conductive pathways formed by carbon black and the formation of larger clusters of CB aggregates in the sample. A more detailed mechanism is described in Section 3.5. The surface of the CB–PLA190 °C ×5 sample is flatter and more homogeneous compared to the CB–PLA200 °C ×5 sample. It can be presumed that the polymer chain in the ×5 at 200 °C sample does not degrade as intensively as in the sample at 190 °C (as confirmed by the MFI measurement; see Section 3.1).

Figure 5d presents the exemplary Raman spectra recorded for the reference sample and the CB–PLA specimens reprocessed five times at 190 °C and 200 °C. Two distinct peaks at about 1350 cm^−1^ and 1600 cm^−1^, associated with the D and G bands, respectively, originate from carbon black presence [46,47,48]. The D band represents disorder or defects in the lattice and corresponds to sp^3^-bonded carbon. The G band relates to the first-order Raman band of all sp^2^-hybridized carbons. It can be seen that the intensity of the carbon peaks increased after filament reprocessing, which can be attributed to the temperature-induced degradation of the PLA matrix. This resulted in a stronger signal from carbon black. In order to evaluate the surface integrity of the composite filaments more precisely, the ratio of the D and G peaks (I_D_/I_G_) was calculated for the reference and the reprocessed samples. Table 4 gathers I_D_/I_G_ parameters for the investigated specimens.

The biggest difference occurs between the reference sample and the reprocessed filaments. There is little difference in the I_D_/I_G_ ratio regarding the reprocessing temperature. An increase in I_D_/I_G_ for reprocessed filaments indicates lower surface integrity, which could be attributed to two factors. Firstly, the fragmentation of the PLA chains and polymer additives due to thermal degradation may lead to a more disordered structure after reprocessing, where some of the carbon black particles become more closely surrounded by degradation-shortened polymer chains while the other ones remain in the form of aggregates. Another factor could be the oxidation of some carbon black particles, which adds heterogeneity to the investigated system.

### 3.4. Electric Conductivity Behavior

The AC conductivity was measured as a function of frequency and possible operation temperature range (0–40 °C) after reprocessing. The measured conductivity, σ, was frequency-independent in the entire range (10 mHz–1 MHz) for the CB–PLA samples before and after reprocessing, showing the domination of DC conductivity. In the temperature range between 10 and 40 °C, the observed conductivity deviation reaches up to only 0.002 S/cm for all samples and can therefore be neglected. Accordingly, we can conclude that the conduction process taking place at these temperatures is not thermally activated. In general, significant changes in the temperature function were not expected and, despite multiple re-melting, DC conductivity is dominant and temperature-independent, indicating an electronic conduction mechanism correlated with the carbon content.

**Figure 6:**
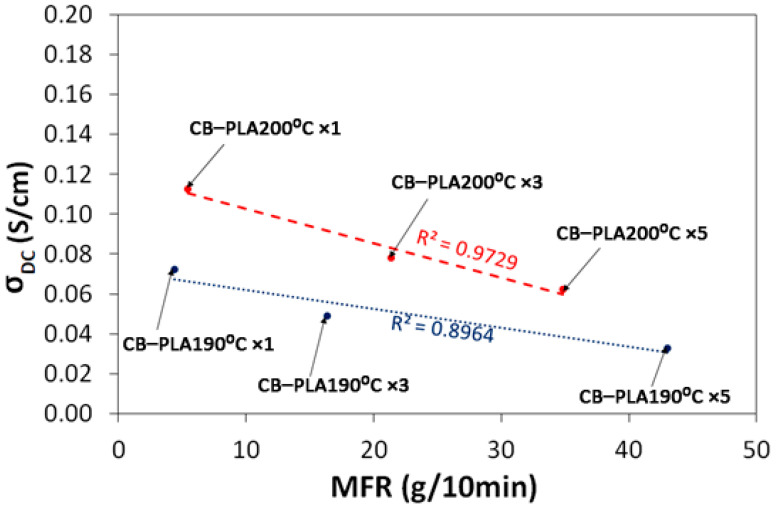
Changes in electrical conductivity as a function of MFR changes for multiple reprocessing cycles of CB–PLA at 190 °C and 200 °C.

The DC conductivity values found for all samples were selected for a frequency of 100 Hz as the one which is a fairly good representative of σ_DC_, and for a temperature of 25 °C as close to the room temperature. The collection is displayed in Figure 6. The CB–PLA reference sample showed a σ_DC_ similar to the CB–PLA200 °C ×1 sample; it was equal to 0.11 S/cm. This presentation clearly shows the effect of reprocessing. Regarding the changes for different samples, one conclusion is that most of the samples (except one CB–PLA200 °C ×1) showed a slight decrease in conductivity after the modification. The successive melts CB–PLA190 °C ×1 >> CB–PLA190 °C ×3 >> CB–PLA190 °C ×5 and, similarly, at 200 °C, show a slow decrease in conductivity. The DC conductivity values for both series were linearly fitted. The fitting results are as follows: y = −0.001x + 0.0716 with R^2^ = 0.8964 for the series re-melted at 190 °C; and y = −0.0017x + 0.1199 with R^2^ = 0.9729 for the series re-melted at 200 °C. However, the effect of increasing the conductivity for higher temperatures of re-melting is interesting. Since all the samples contain the same amount of carbon black filler, which is the decisive factor dictating the conduction process, the decrease in conductivity can be correlated with the matrix effect. The reduction in the viscosity of the tested material by re-melting processes can reduce the number of available percolation paths, which reduces conductivity.

### 3.5. Electrochemical Activity of the Reprocessed CB–PLA Filament

**Figure 7:**
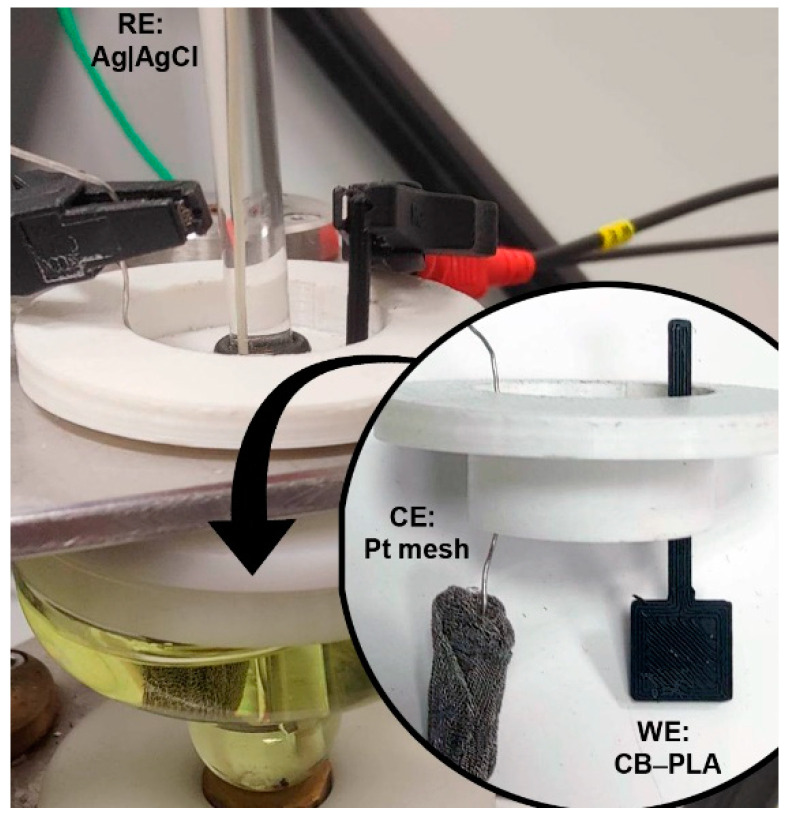
The electrochemical setup containing the CB–PLA working electrode, printed after three reprocessing cycles of the filament.

Last but not least, we studied the effect of multiple CB–PLA reprocessing on the electrochemical response. The previously described decline in rheological parameters and excessive fluidity of the composite leads to a significant loss of adhesion between consecutive layers and the leakage of the electrolyte. Changing the design to an electrode with an electrical contact did not give good results either. The electrical contact broke when it was connected to the potentiostat. Thus, the effective formation of 3D-printed electrodes was impossible after three or more CB–PLA reprocessing cycles. The CB–PLA electrode printed after three reprocessing cycles is shown in Figure 7. As a result, an effective comparison of the electrochemical characteristics of reprocessed CB–PLA electrodes compelled the use of hydraulic pressing to prepare coherent samples for electrochemical testing. The hydraulic pressing should be considered an additional processing cycle.

**Figure 8:**
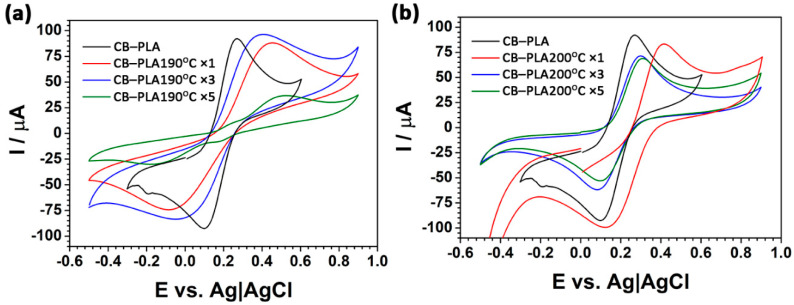
CV measurements of commercial filaments processed ×1, ×2, ×4 and ×6; (**a**) PP processed at 190 °C, (**b**) PP processed at 200 °C. Electrolyte: 1 mM [Fe(CN)_6_]^3−/4−^ + 0.1 M KCl, V_s_ = 100 mV/s.

The voltammograms (carried out at 100 mV/s) presented in Figure 8 allow observing how reprocessing temperature affects the reaction kinetics by [Fe(CN)_6_]^3−/4−^. One must note that the mechanism of the charge transfer by the CB–PLA electrode, as studied by the CV, is quite complex in nature and only occurs at the conductive carbon filler surface, not within the polymer matrix. Thus, the electrode characteristics strictly depend on the distribution homogeneity of the filler at the electrode surface. If good dispersion is achieved, local diffusion fields surrounding each CB particle are heavily overlapping and the response is similar to that of the flat electrode.

The effect of multiple reprocessing at an elevated temperature is expected to affect CB aggregate formation, the lowering of the homogeneity of CB dispersion, and the percolation paths formation within the polymer matrix.

The registered [Fe(CN)_6_]^3−/4−^ oxidation and reduction currents systematically decrease with the number of reprocessing cycles, allowing to draw the conclusions regarding the congenial values of the electroactive surface area (ESA). The [Fe(CN)_6_]^3−/4−^ oxidation current I_a,p_ drops down after the fifth reprocessing cycle by 60% at 190 °C, and by 25% at 200 °C. While the ESA decreases even after the first remelting process at 200 °C, its eventual drop is lower compared to the reprocessing at 190 °C. The observed behavior is the result of the decreased number of percolation paths due to the combined effect of a higher CB dispersion when processing at 190 °C and a lower adhesion in between the CB–PLA layers. It is observed that reprocessing declines material conductivity. Considering the decreased number of percolation paths, part of the electrode surface might be electrically cut off from the electrode volume, effectively reducing ESA. The remaining CB particle agglomerates at the electrode surface are characterized by individual diffusion layers, spherical in nature, leading to a partially blocked electrode effect [49]. On the other hand, a rapid I_a,p_ decrease at 200 °C may be a consequence of a lower number of voids in the material [9], also resulting in the lower electrochemical activation efficiency of the CB–PLA surfaces. The lower saponification surface uncovers the conductive CB filler less effectively, decreasing ESA development.

A study of the reference CB–PLA sample shows peak-to-peak separation ΔE_p_ ~165 mV, comparable to the previously reported for CB–PLA 3D prints [31]. It appears that even the first reprocessing at 190 °C lowers the reversibility of the redox process. The ΔE_p_ ~526 mV significantly exceeds the theoretical value for the one-electron–one-step reversible reaction [50], yet the relative oxidation/reduction peak current symmetry hints at the quasi-reversibility of the process. A significant decline in the electrochemical response is visible when surpassing the fifth reprocessing cycle at 190 °C, as it is marked by a ΔE_p_ increase up to 700 mV, as shown in Table 5. The ΔE_p_ after three and five remelting cycles of the CB–PLA composite are 212 and 206 mV, respectively. These values still lie in the applicability range for the determination of the heterogeneous rate constant k^0^ for the one-step–one-electron reaction using the Nicholson approach [42,51]. Considering this observation, the drop in electron transfer reversibility seems to be affected primarily by the time of the process rather than the temperature.

There are several reasons why thermal treatment and polymer reprocessing may affect the reversibility of the charge transfer process, displayed in particular for the redox species characterized by inner-sphere electron transfer, such as [Fe(CN)_6_]^3−/4−^. Here, the electrode kinetics strongly relies on the presence of oxidized carbon species or the nonspecific adsorption at the electrode surface [52]. CB–PLA reprocessing in the air may lead to CB particles oxidation and termination by hydroxyl or carboxyl species [17]. Moreover, thermal degradation of the polymer chains may hinder the saponification process, essential for successful electrochemical activation [31].

**Figure 9:**
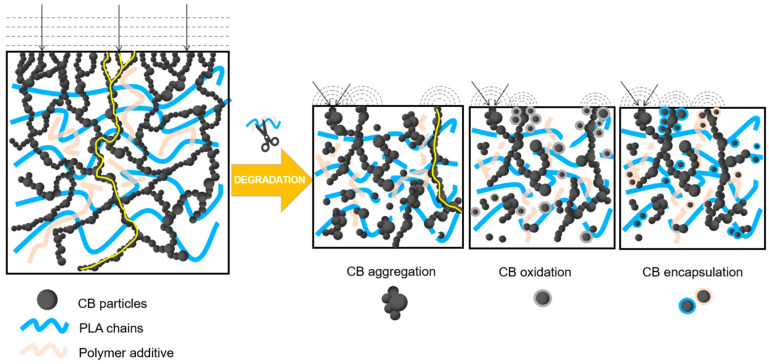
Schematic degradation mechanism of multiple reprocessed CB–PLA.

Considering the above data, the scheme of the degradation mechanism during the regeneration of CB–PLA composites is shown in Figure 9. The structure of the untreated PLA–CB sample is shown on the left. We can see a uniform distribution of entangled CB agglomerates, i.e., numerous percolation pathways responsible for the conductivity of the sample (yellow line). In the reference material, there are no carbon black aggregates, and the structure is ordered. After the melt processing of the sample, there are structural changes in the material. As can be seen, there are several possible transformation paths. Each of them is related to the thermal degradation of PLA and the polymer additive used by the filament manufacturer, which simultaneously improves the dispersion of carbon black in the polymer matrix. The polymer chains break into shorter polymer fragments with lower molecular weight under the influence of high temperature and residence time in the plastometer barrel. As a result of material degradation, the percolation pathways are rearranged. Their occurrence changes, but they do not disappear. This leads to the formation of CB aggregates. The consequence of such a mechanism is a reduction in the number of conductive pathways in the material. Possible mechanisms that may also occur are the oxidation of the carbon black particles or their encapsulation, i.e., the surrounding of the CB particles by polymer chains, which may lead to a reduction in their participation in the conductivity process.

Recently, Gao et al. [53] presented interesting studies about the effect of thermal treatment (200 °C, 2 h) on the electrical and rheological properties of poly(methyl methacrylate) and polystyrene composites filled with carbon black. The main finding showed that annealing treatments resulted in the decrease in the electrical conductivity of conductive polymer composites, which can be attributed to the breakdown of the unstable conductive pathways and the formation of secondary CB aggregates, which is in agreement with our results.

## 4. Conclusions

In this work, the effect of multiple reprocessing (up to five cycles) of conductive PLA 3D-printing filament with carbon black filler was investigated. The effects of the processing temperature on the melt flow index, thermal properties, microstructure, electrical and electrochemical properties were characterized. Based on the results obtained, the main findings are pointed out below:

- Multiple reprocessing cycles of commercial conductive CB–PLA resulted in the degradation of the polymer matrix, regardless of the processing temperature (when studied at 190 and 200 °C);
- Decomposition temperatures are significantly affected by the reprocessing number, where T_−2%_ is 15.7 °C (190 °C) and 11.6 °C (200 °C) lower compared to the reference sample, respectively;
- A lower processing temperature increases the residence time of CB–PLA in the extruder’s barrel (by 24.5% for the first cycle), which may cause, under specific conditions, a higher degradation even at lower temperatures;
- The further the degradation progresses, the more reduced the electrical conductivity of the CB–PLA composite is, down to 0.06 S/cm (1.7 times decrease) and 0.03 S/cm (3.7 times decrease) when ×5 reprocessed at 190 and 200 °C, respectively. This phenomenon is related to the combined effects of CB dispersibility and PLA chain cleavage;
- At the same time, the electrochemical properties, manifested by the reversibility of the redox process and the electrochemical surface area, are also affected due to the competitive filler oxidation and CB aggregate formation, leading to partially blocked electrode effects. The [Fe(CN)_6_]^3−/4−^ redox currents drop by 60% at 190 °C, and 25% at 200 °C, while ΔE_p_ increases from 165 to ~210 mV at 200 °C, and above 700 mV at 190 °C;
- The plastometer can be successfully applied in the preparation of 3D-printable filaments, which might be very useful in the studies on novel and expensive fillers.

It should be highlighted that the measured electrical and electrochemical properties are highly dependent on the methodology used. Therefore, future research in this field should consider the development of standardized protocols. This approach allows for the comparison of the results obtained by independent research groups. Moreover, this aspect is extremely important considering the current trends focused on the preparation of novel conductive filaments with well-defined compositions dedicated to special applications, e.g., electrochemical sensors.

## Figures and Tables

**Table 1:** Decomposition temperatures as a function of weight losses, different maximum peaks and char residue determined for pure and recycled conductive PLA 3D filament.

Sample Code	T_−2%_ (°C)	T_−5%_ (°C)	T_−10%_ (°C)	T_−50%_ (°C)	T_max1_ (°C)	T_max2_ (°C)	Char Residue at 800 °C (%)
CB–PLA	327.2	337.5	345.8	372.7	366.2	458.7	21.6
CB–PLA190 °C ×1	321.9	334.7	343.9	371.5	367.0	459.1	22.4
CB–PLA190 °C ×3	316.0	329.9	340.0	369.4	365.3	457.4	21.0
CB–PLA190 °C ×5	311.5	326.2	336.9	367.6	363.2	458.0	22.3
CB–PLA200 °C ×1	324.9	336.9	345.4	371.9	367.4	460.2	21.7
CB–PLA200 °C ×3	320.1	333.2	342.7	370.2	365.8	460.7	22.7
CB–PLA200 °C ×5	315.3	330.9	341.2	370.2	366.2	459.2	20.6

**Table 2:** DSC results for processed commercial CB–PLA filaments.

Sample Code	T_g_ (°C)	T_m_ (°C)	ΔH_cc_ (J/g)	ΔH_m_ (J/g)
CB–PLA	60.2	154.2	2.1	26.2
CB–PLA190 °C ×1	59.8	157.9	19.0	20.4
CB–PLA190 °C ×3	58.8	157.0	26.5	27.0
CB–PLA190 °C ×5	58.7	157.0	25.1	26.1
CB–PLA200 °C ×1	59.1	153.4	20.9	21.3
CB–PLA200 °C ×3	59.4	157.2	21.1	21.5
CB–PLA200 °C ×5	59.9	157.5	22.9	23.6

**Table 4:** Values of I_D_/I_G_ ratio calculated from the Raman spectra for CB–PLA samples.

Sample Code	I_D_/I_G_ Parameter
CB–PLA	0.85
CB–PLA190 °C ×5	0.94
CB–PLA200 °C ×5	0.98

**Table 5:** Values of ΔE and I_a_ for CV scans at 100 mV/s after multiple reprocessing of PP.

Sample Code	ΔE_p_/mV	I_a,p_/µA	k^0^_obs_/cm/s
PP	165	92	2.25·10^−3^
PP190 °C ×1	526	88	-
PP190 °C ×3	418	96	-
PP190 °C ×5	702	37	-
PP200 °C ×1	270	83	-
PP200 °C ×3	212	72	8.99·10^−4^
PP200 °C ×5	206	69	2.25·10^−3^

## Data Availability

Not applicable.

## References

[1] (2012). Standard Terminology for Additive Manufacturing Technologies.

[2] Gjokaj V., Crump C., Wright B., Chahal P. Direct Printing of Antennas on Large 3D Printed Plastic Structures. Proceedings of the 2020 IEEE 70th Electronic Components and Technology Conference (ECTC).

[3] Joshi S.C., Sheikh A.A. (2015). 3D Printing in Aerospace and Its Long-Term Sustainability. Virtual Phys. Prototyp..

[4] Sacco E., Moon S.K. (2019). Additive Manufacturing for Space: Status and Promises. Int. J. Adv. Manuf. Technol..

[5] Walker J.S., Arnold J., Shrestha C., Smith D. (2020). Antibacterial Silver Submicron Wire-Polylactic Acid Composites for Fused Filament Fabrication. RPJ.

[6] Wang C., Huang W., Zhou Y., He L., He Z., Chen Z., He X., Tian S., Liao J., Lu B. (2020). 3D Printing of Bone Tissue Engineering Scaffolds. Bioact. Mater..

[7] Gu Q., Hao J., Lu Y., Wang L., Wallace G.G., Zhou Q. (2015). Three-Dimensional Bio-Printing. Sci. China Life Sci..

[8] Lazarus N., Bedair S.S. (2021). Creating 3D Printed Sensor Systems with Conductive Composites. Smart Mater. Struct..

[9] Kwok S.W., Goh K.H.H., Tan Z.D., Tan S.T.M., Tjiu W.W., Soh J.Y., Ng Z.J.G., Chan Y.Z., Hui H.K., Goh K.E.J. (2017). Electrically Conductive Filament for 3D-Printed Circuits and Sensors. Appl. Mater. Today.

[10] Flowers P.F., Reyes C., Ye S., Kim M.J., Wiley B.J. (2017). 3D Printing Electronic Components and Circuits with Conductive Thermoplastic Filament. Addit. Manuf..

[11] Allen N.S., Pena J.M., Edge M., Liauw C.M. (2000). Behaviour of Carbon Black Pigments as Excited State Quenchers in LDPE. Polym. Degrad. Stab..

[12] Mosnáčková K., Danko M., Šišková A., Falco L.M., Janigová I., Chmela Š., Vanovčanová Z., Omaníková L., Chodák I., Mosnáček J. (2017). Complex Study of the Physical Properties of a Poly(Lactic Acid)/Poly(3-Hydroxybutyrate) Blend and Its Carbon Black Composite during Various Outdoor and Laboratory Ageing Conditions. RSC Adv..

[13] Azura A.R., Leow S.L. (2019). Effect of Carbon Black Loading on Mechanical, Conductivity and Ageing Properties of Natural Rubber Composites. Mater. Today Proc..

[14] Liu M., Horrocks A.R. (2002). Effect of Carbon Black on UV Stability of LLDPE Films under Artificial Weathering Conditions. Polym. Degrad. Stab..

[15] D’Urso L., Acocella M., Guerra G., Iozzino V., De Santis F., Pantani R. (2018). PLA Melt Stabilization by High-Surface-Area Graphite and Carbon Black. Polymers.

[16] Batista Deroco P., Campanhã Vicentini F., Fatibello-Filho O. (2015). An Electrochemical Sensor for the Simultaneous Determination of Paracetamol and Codeine Using a Glassy Carbon Electrode Modified with Nickel Oxide Nanoparticles and Carbon Black. Electroanalysis.

[17] Glowacki M.J., Cieslik M., Sawczak M., Koterwa A., Kaczmarzyk I., Jendrzejewski R., Szynkiewicz L., Ossowski T., Bogdanowicz R., Niedzialkowski P. (2021). Helium-Assisted, Solvent-Free Electro-Activation of 3D Printed Conductive Carbon-Polylactide Electrodes by Pulsed Laser Ablation. Appl. Surf. Sci..

[18] Hamzah H.H., Shafiee S.A., Abdalla A., Patel B.A. (2018). 3D Printable Conductive Materials for the Fabrication of Electrochemical Sensors: A Mini Review. Electrochem. Commun..

[19] Rocha D.P., Squissato A.L., da Silva S.M., Richter E.M., Munoz R.A.A. (2020). Improved Electrochemical Detection of Metals in Biological Samples Using 3D-Printed Electrode: Chemical/Electrochemical Treatment Exposes Carbon-Black Conductive Sites. Electrochim. Acta.

[20] Cardoso R.M., Castro S.V.F., Silva M.N.T., Lima A.P., Santana M.H.P., Nossol E., Silva R.A.B., Richter E.M., Paixão T.R.L.C., Muñoz R.A.A. (2019). 3D-Printed Flexible Device Combining Sampling and Detection of Explosives. Sens. Actuators B Chem..

[21] Woern A., Byard D., Oakley R., Fiedler M., Snabes S., Pearce J. (2018). Fused Particle Fabrication 3-D Printing: Recycled Materials’ Optimization and Mechanical Properties. Materials.

[22] Zhao P., Rao C., Gu F., Sharmin N., Fu J. (2018). Close-Looped Recycling of Polylactic Acid Used in 3D Printing: An Experimental Investigation and Life Cycle Assessment. J. Clean. Prod..

[23] Mikula K., Skrzypczak D., Izydorczyk G., Warchoł J., Moustakas K., Chojnacka K., Witek-Krowiak A. (2021). 3D Printing Filament as a Second Life of Waste Plastics—A Review. Environ. Sci Pollut. Res..

[24] Lanzotti A., Martorelli M., Maietta S., Gerbino S., Penta F., Gloria A. (2019). A Comparison between Mechanical Properties of Specimens 3D Printed with Virgin and Recycled PLA. Procedia CIRP.

[25] Anderson I. (2017). Mechanical Properties of Specimens 3D Printed with Virgin and Recycled Polylactic Acid. 3D Print. Addit. Manuf..

[26] Hong J.-H., Yu T., Park S.-J., Kim Y.-H. (2020). Repetitive Recycling of 3D Printing PLA Filament as Renewable Resources on Mechanical and Thermal Loads. Int. J. Mod. Phys. B.

[27] Gil Muñoz V., Muneta L.M., Carrasco-Gallego R., de Juanes Marquez J., Hidalgo-Carvajal D. (2020). Evaluation of the Circularity of Recycled PLA Filaments for 3D Printers. Appl. Sci..

[28] Kotsilkova R., Petrova-Doycheva I., Menseidov D., Ivanov E., Paddubskaya A., Kuzhir P. (2019). Exploring Thermal Annealing and Graphene-Carbon Nanotube Additives to Enhance Crystallinity, Thermal, Electrical and Tensile Properties of Aged Poly(Lactic) Acid-Based Filament for 3D Printing. Compos. Sci. Technol..

[29] Kalinke C., de Oliveira P.R., Neumsteir N.V., Henriques B.F., de Oliveira Aparecido G., Loureiro H.C., Janegitz B.C., Bonacin J.A. (2022). Influence of Filament Aging and Conductive Additive in 3D Printed Sensors. Anal. Chim. Acta.

[30] Crespo-Miguel J., Garcia-Gonzalez D., Robles-Muñoz G., Hossain M., Martinez-Tarifa J.M., Arias Á. (2022). Thermo-Electro-Mechanical Aging and Degradation of Conductive 3d Printed Polymers. SSRN J..

[31] Koterwa A., Kaczmarzyk I., Mania S., Cieślik M., Tylingo R., Ossowski T., Bogdanowicz R., Niedziałkowski P., Ryl J. (2022). The Role of the Electrolysis and Enzymatic Hydrolysis in the Enhancement of the Electrochemical Properties of 3D-Printed Carbon Black/Poly(Lactic Acid) Structures. Appl. Surf. Sci..

[32] Seifali Abbas-Abadi M., Nekoomanesh Haghighi M., Yeganeh H., Bozorgi B. (2013). The Effect of Melt Flow Index, Melt Flow Rate, and Particle Size on the Thermal Degradation of Commercial High Density Polyethylene Powder. J. Anal. Calorim..

[33] Singh B., Kumar R., Chohan J.S., Singh S., Pruncu C.I., Scutaru M.L., Muntean R. (2021). Investigations on Melt Flow Rate and Tensile Behaviour of Single, Double and Triple-Sized Copper Reinforced Thermoplastic Composites. Materials.

[34] Barczewski M., Matykiewicz D., Andrzejewski J., Skórczewska K. (2016). Application of Waste Bulk Moulded Composite (BMC) as a Filler for Isotactic Polypropylene Composites. J. Adv. Res..

[35] Wang S., Capoen L., D’hooge D.R., Cardon L. (2018). Can the Melt Flow Index Be Used to Predict the Success of Fused Deposition Modelling of Commercial Poly(Lactic Acid) Filaments into 3D Printed Materials?. Plast. Rubber Compos..

[36] Carrasco F., Santana Pérez O., Maspoch M. (2021). Kinetics of the Thermal Degradation of Poly(Lactic Acid) and Polyamide Bioblends. Polymers.

[37] Stefano J.S., e Silva L.R.G., Janegitz B.C. (2022). New Carbon Black-Based Conductive Filaments for the Additive Manufacture of Improved Electrochemical Sensors by Fused Deposition Modeling. Microchim. Acta.

[38] Katseli V., Thomaidis N., Economou A., Kokkinos C. (2020). Miniature 3D-Printed Integrated Electrochemical Cell for Trace Voltammetric Hg(II) Determination. Sens. Actuators B Chem..

[39] Omastová M., Podhradská S., Prokeš J., Janigová I., Stejskal J. (2003). Thermal Ageing of Conducting Polymeric Composites. Polym. Degrad. Stab..

[40] Voet A. (1981). Temperature Effect of Electrical Resistivity of Carbon Black Filled Polymers. Rubber Chem. Technol..

[41] Sirjani E., Cragg P.J., Dymond M.K. (2019). Glass Transition Temperatures, Melting Temperatures, Water Contact Angles and Dimensional Precision of Simple Fused Deposition Model 3D Prints and 3D Printed Channels Constructed from a Range of Commercially Available Filaments. Chem. Data Collect..

[42] Lavagnini I., Antiochia R., Magno F. (2004). An Extended Method for the Practical Evaluation of the Standard Rate Constant from Cyclic Voltammetric Data. Electroanalysis.

[43] Kim H., Lee S. (2020). Characterization of Electrical Heating of Graphene/PLA Honeycomb Structure Composite Manufactured by CFDM 3D Printer. Fash Text.

[44] da Silva T.F., Menezes F., Montagna L.S., Lemes A.P., Passador F.R. (2019). Preparation and Characterization of Antistatic Packaging for Electronic Components Based on Poly(Lactic Acid)/Carbon Black Composites. J. Appl. Polym. Sci..

[45] Guo J., Tsou C.-H., Yu Y., Wu C.-S., Zhang X., Chen Z., Yang T., Ge F., Liu P., Guzman M.R.D. (2021). Conductivity and Mechanical Properties of Carbon Black-Reinforced Poly(Lactic Acid) (PLA/CB) Composites. Iran. Polym. J..

[46] Ferrari A.C., Basko D.M. (2013). Raman Spectroscopy as a Versatile Tool for Studying the Properties of Graphene. Nat. Nanotechnol..

[47] Ferrari A.C., Meyer J.C., Scardaci V., Casiraghi C., Lazzeri M., Mauri F., Piscanec S., Jiang D., Novoselov K.S., Roth S. (2006). Raman Spectrum of Graphene and Graphene Layers. Phys. Rev. Lett..

[48] Graf D., Molitor F., Ensslin K., Stampfer C., Jungen A., Hierold C., Wirtz L. (2007). Raman Imaging of Graphene. Solid State Commun..

[49] Davies T.J., Banks C.E., Compton R.G. (2005). Voltammetry at Spatially Heterogeneous Electrodes. J. Solid State Electrochem..

[50] Bard A.J., Faulkner L.R. (2001). Electrochemical Methods: Fundamentals and Applications.

[51] Ryl J., Burczyk L., Zielinski A., Ficek M., Franczak A., Bogdanowicz R., Darowicki K. (2019). Heterogeneous Oxidation of Highly Boron-Doped Diamond Electrodes and Its Influence on the Surface Distribution of Electrochemical Activity. Electrochim. Acta.

[52] Chen P., Fryling M.A., McCreery R.L. (1995). Electron Transfer Kinetics at Modified Carbon Electrode Surfaces: The Role of Specific Surface Sites. Anal. Chem..

[53] Gao Q., Liu J., Liu X. (2021). Electrical Conductivity and Rheological Properties of Carbon Black Based Conductive Polymer Composites Prior to and after Annealing. Polym. Polym. Compos..

